# Sharing Happy Stories Increases Interpersonal Closeness: Interpersonal Brain Synchronization as a Neural Indicator

**DOI:** 10.1523/ENEURO.0245-21.2021

**Published:** 2021-11-18

**Authors:** Enhui Xie, Qing Yin, Keshuang Li, Samuel A. Nastase, Ruqian Zhang, Ning Wang, Xianchun Li

**Affiliations:** 1 Shanghai Key Laboratory of Mental Health and Psychological Crisis Intervention, Affiliated Mental Health Center (ECNU), School of Psychology and Cognitive Science, East China Normal University, Shanghai 200062, China; 2 Princeton Neuroscience Institute, Princeton University, Princeton, NJ 08544

**Keywords:** emotion, interpersonal brain synchronization, interpersonal closeness, sharing stories

## Abstract

Our lives revolve around sharing emotional stories (i.e., happy and sad stories) with other people. Such emotional communication enhances the similarity of story comprehension and neural across speaker-listener pairs. The theory of Emotions as Social Information Model (EASI) suggests that such emotional communication may influence interpersonal closeness. However, few studies have examined speaker-listener interpersonal brain synchronization (IBS) during emotional communication and whether it is associated with meaningful aspects of the speaker-listener interpersonal relationship. Here, one speaker watched emotional videos and communicated the content of the videos to 32 people as listeners (happy/sad/neutral group). Both speaker and listeners’ neural activities were recorded using EEG. After listening, we assessed the interpersonal closeness between the speaker and listeners. Compared with the sad group, sharing happy stories showed a better recall quality and a higher rating of interpersonal closeness. The happy group showed higher IBS in the frontal cortex and left temporoparietal cortex than the sad group. The relationship between frontal IBS and interpersonal closeness was moderated by sharing happy/sad stories. Exploratory analysis using support vector regression (SVR) showed that the IBS could also predict the ratings of interpersonal closeness. These results suggest that frontal IBS could serve as an indicator of whether sharing emotional stories facilitate interpersonal closeness. These findings improve our understanding of emotional communication among individuals that guides behaviors during interpersonal interactions.

## Significance Statement

Despite extensive research on interpersonal communication, little is known about emotional communication (happy/sad) between speaker and listener and whether these two types of emotional communication involve differential neurocognitive mechanisms from a brain-to-brain perspective. We address these questions from the perspective of the brain-to-brain approach and suggest that these two types of emotional communication are associated with differential interpersonal brain synchronization (IBS), in particular, subserved by the prefrontal region. Our findings shed light on the effect of sharing emotional (happy/sad) stories on interpersonal closeness and suggest that frontal IBS could serve as an indicator of whether sharing emotional (happy/sad) stories facilitate interpersonal closeness.

## Introduction

Sharing specific stories with another person plays an important role in social interaction. Sharing stories is a way for people to organize and convey their thoughts (Willems et al., 2020), a way to enhance people’s ability to predict themselves and each other (Pickering and Garrod, 2013), and a social practice promoting the formation of collective memory (Hirst and Echterhoff, 2012). Maswood and Rajaram (2019) have demonstrated that sharing stories is accompanied by expressing emotional meaning. Sharing an emotional story is a social interaction during which emotional brain states are transmitted between speaker and listeners (Hasson et al., 2012; Chen et al., 2017; Zadbood et al., 2017). For example, Zadbood et al. (2017) demonstrated that the listener mentally reconstructs the episodes of a story when listening to a speaker’s recollection of an audiovisual movie, even if the listener did not watch the movie before. Nonetheless, relatively little is known about the effect of emotional communication on interpersonal relationship.

The theory of Emotions as Social Information Model (EASI) proposes that expressing emotional information is a social signal in interpersonal interaction (Van Kleef, 2009) and thus may increase interpersonal closeness. The listener receives both conscious and unconscious social cues from the speaker’s emotional expressions and the listener can regulate their emotional state to increase synchrony of emotional states with the speaker (Hari et al., 2015). This alignment may influence the interpersonal closeness of the speaker and listener during emotion-related interaction. Previous studies have provided evidence that the expression of emotional information can promote mutual understanding, strengthen interpersonal communication, and promote social connections (Nummenmaa et al., 2012; Dubois et al., 2014; Smirnov et al., 2019).

A stream of research has suggested that sharing both happy and sad stories may be critical to building a good interpersonal relationship (Isgett and Fredrickson, 2015; Shoham et al., 2016). Sharing happy stories can promote the attainment of desirable outcomes, such as obtaining social gratification from interpersonal interactions and strengthening interpersonal bonds (Fredrickson, 2001; Isgett and Fredrickson, 2015) by efficiently shaping a positive image to others (Ranzini and Hoek, 2017; Johnson and Ranzini, 2018). Sharing sad stories can enhance positive impressions and build close relationships based on powerful negative biases (Baumeister et al., 2001; Rozin and Royzman, 2001; Vaish et al., 2008; Fessler et al., 2015; Shoham et al., 2016). Although sharing happy and sad stories may both facilitate the interpersonal closeness, individuals seem to prefer to share the positive events in the story and suppress the negative ones (Gillath et al., 2005; Piotroski et al., 2015). When the speaker is sharing happy stories, the listener may experience feelings of pleasure, and even a sense of well-being; however, when the speaker is sharing sad stories, the listener may experience a sad feeling (Hanley et al., 2017). Such differences in emotional states are associated with differences in behavioral, physiological, and cognitive components (Anderson and Adolphs, 2014), and thus positive emotional state matching between speaker and listener may facilitate interpersonal relationships effectively than negative emotional communication. All of the above suggested that sharing happy stories may play a more important role in enhancing interpersonal closeness than sharing sad stories. However, the definite behavioral effect of sharing different emotional stories (happy/sad) on influencing interpersonal closeness remains unevaluated.

Interpersonal brain synchronization (IBS) can be a neuromarker of various interpersonal relationships during emotional communication (Stephens et al., 2010; Nummenmaa et al., 2012). Previous neuroimaging studies have indicated that sharing emotional stories causes individuals to be “on the same page” neurally (Dikker et al., 2014). The higher similarity of the neural responses in speaker-listener dyads has been associated with an increased shared interpretation of the narrative (Zadbood et al., 2017; Nguyen et al., 2019). Several studies have observed alignment of neural responses between the speaker and listener in a network of high-level cortical regions typically attributed to the prefrontal cortex (PFC) activity during such an emotional communication process (Stephens et al., 2010; Silbert et al., 2014; Zadbood et al., 2017). Further, neuroimaging results suggested that brain activity in the θ band was correlated with emotion, memory encoding, and information transmitting (Klimesch et al., 1996; Ding, 2010; Zheng et al., 2012; Symons et al., 2016). However, the neural process of sharing different emotional stories (happy/sad) on influencing interpersonal closeness remains unclear. Based on previous studies, we expected IBS as a neural indicator to uncover the neural mechanism of sharing emotional stories and interpersonal closeness during interpersonal interaction and expected to observe the strongest closeness-related IBS in the θ band mainly in the PFC.

The present study aims to provide behavioral and neural evidence for evaluating the effect of sharing emotional stories on influencing interpersonal closeness within the theoretical framework of EASI. Building on previous studies (Niedenthal and Setterlund, 1994; Ribeiro et al., 2019), the present study manipulates the valence of emotional stories (Happy vs Sad) to reveal the effects of sharing emotional stories on interpersonal closeness. The neural mechanism of sharing emotional stories influencing interpersonal closeness is investigated from the perspective of brain-to-brain coupling. On the behavioral level, we expected that sharing emotional stories would influence interpersonal closeness. Specifically, we hypothesized that sharing happy stories will increase interpersonal closeness more effectively than sharing sad ones. On the neural level, we expected that sharing happy stories will yield higher IBS than sharing sad stories in the θ band mainly in the PFC. Finally, we hypothesized that enhanced IBS will mediate the effect of sharing emotional stories on influencing interpersonal closeness.

## Materials and Methods

### Participants

A total of 32 participants (age: 21.3 ± 2.4 years, 16 females) were enrolled as listeners in the present study. Specifically, all the listeners were randomly assigned to listen to the happy stories from a competent speaker as speaker-listener dyads (15 listeners in the happy group) or the sad stories from the same competent speaker as speaker-listener dyads (17 listeners in the sad group).

One competent speaker (female, 19 years of age) was initially determined in a comprehension test. During this comprehension test, an independent sample of *n* = 10 participants (age: 22.1 ± 2.2 years, eight females) were asked to watch the emotional videos and narrate each video. The narrations were recorded and qualitatively assessed with the understanding of the stories and the accuracy of the emotion in the stories by three independent raters. Suggested items to consider were (1) understanding of the stories, (2) expression of the episodes, (3) the number of scenes remembered, (4) details provided, and (5) the accuracy of emotion in the stories. They reported a score for each participant across the three raters. The brain data for the selected speaker were manually inspected for quality, and the data from the other speakers are not further analyzed here (the rating sheet made is provided in Table 1).

**Table 1:** Speakers’ comprehension test score

Speaker number	Happy	Neutral	Sad	Average score
101	12	13	10	11.67
102	11	21	18	16.67
103	21	18	25	21.33
104	16	16	20	17.33
105	19	10	25	18.00
106	18	17	12	15.67
107	25	23	26	24.67
108	20	16	12	16.00
109	18	19	15	17.33
110	10	19	11	13.33

The rater has included an overall comprehension level out of 10 and the total score for each subject was out of 30.

All participants provided written informed consent. The study had full ethical approval by the University Committee on Human Research Protection (UCHRP; HR 403–2019).

### Stimuli

The stimuli consisted of a total of three videos (happy video, sad video, and neutral video). The present study used three audiovisual movies, excerpts from the episodes of happy video (Hail the Judge, ∼5 min in length), sad video (Root and Branches, ∼7 min in length), and neutral video (natural scenes, ∼6 min in length). These videos were chosen to have similar levels of production quality. Further, to assess the valence and the arousal of three videos, 10 raters (age: 20.5 ± 1.6 years, five females) were asked to identify the emotional valence of the videos (happy, neutral, or sad) and their emotional arousal on a 0–9 scale. Moreover, the 10 raters were required to rate the amount of social content and vividness (scale ranging from 1 to 9) on separate nine-point Likert scales. The raters reported a comparative evaluation of the arousal, the amount of social content, and vividness among the happy, neutral, and sad videos. Importantly, there were no significant differences in some ways (emotional arousal, *F*_(2,29)_ = 1.53, *p *>* *0.05, the amount of social content, *F*_(2,29)_ = 1.32, *p *>* *0.05, and vividness, *F*_(2,29)_ = 0.65, *p *>* *0.05) attributes between the happy, neutral, and sad videos. Therefore, we gave more evidence that the minimal baseline differences were demonstrated between the three stimuli.

**Figure 1.**
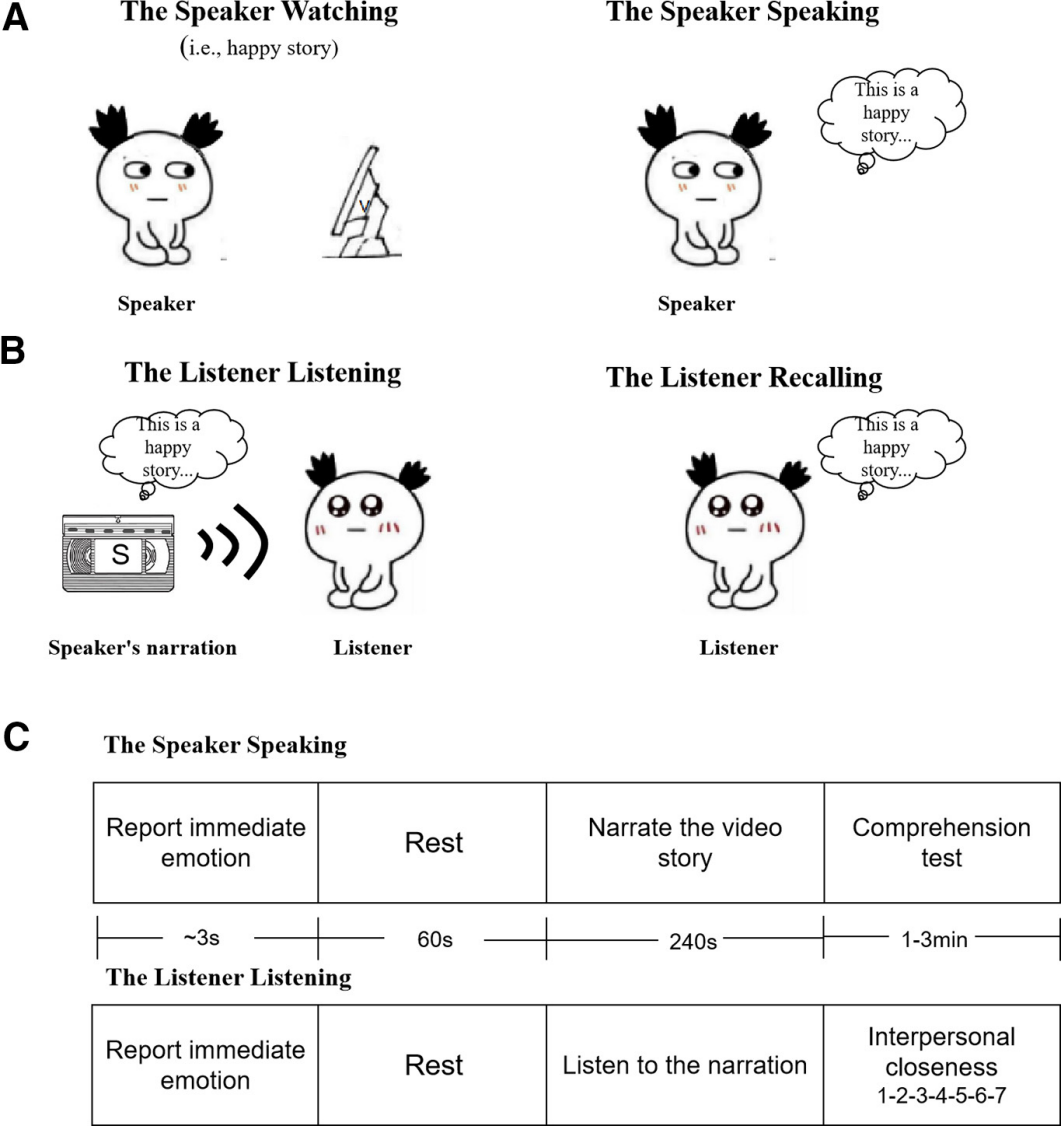
Experimental design. ***A***, Speaker design. The speaker was invited to watch an emotional video and shared the stories in the video by narrating. ***B***, Listener design. The listener was asked to listen to the story of the video through the speaker’s narration and allowed to recall the story which the speaker shared. ***C***, The task in The Speaker Speaking and The Listener Listening. The specific procedure of sharing stories.

Each listener received two-story stimuli, one neutral and one happy or sad. The recall duration of each emotional spoken recall recording was the same. The spoken recall recording of the happy story was 4 min, comprising 400 words; the spoken recall recording of the sad story was 4 min, comprising 420 words; and the spoken recall recording of the neutral story was 4 min, comprising 400 words. Audio recordings were obtained from each speaker who watched and recounted the two videos (one neutral and one happy/sad) with EEG recording. The listener listened to two corresponding audio recordings (Fig. 1*B*).

### Procedures

The experimental procedures consisted of a resting-state phase and a task phase for both the speaker and the listener sessions. The speaker and the listener performed their tasks separately. In all experimental stages, the neural activity of the speaker and the listener was recorded with EEG. During the resting-state phase (60 s), participants were instructed to relax while keeping their eyes closed without falling asleep and to avoid excessive head motion. For each dyad, an initial resting-state session served as a baseline.

The task phase included two main sessions. In the first session (the speaker session), first, the speaker participants were asked to watch the happy, sad, and neutral videos (Fig. 1*A*, Speaker Watching); second, the speakers were asked to verbally narrate the stories in the videos and their narrations were recorded (Fig. 1*A*, Speaker Speaking). The speaker participants’ brain activity was recorded using EEG during speaking. In the second session (the listener session), 32 listeners were invited to listen to the emotional (happy/sad) and neutral stories recordings (The Listener Listening; see Fig. 1*B*), which from the competent speaker who was chosen in the comprehension test (Fig. 1*C*), and recall the corresponding recordings (The Listener Recalling; see Fig. 1*B*).

To control the confounding effects of between-group differences in mood, all listeners were required to report their emotional state immediately before listening. The happy group received happy stories from the competent speaker’s recording, whereas the sad group received sad stories from the competent speaker’s recording. Moreover, sharing neutral stories served as a baseline for sharing emotional performance and therefore it was reasoned that this condition should be performed before the happy or sad condition. To determine the effect of sharing emotional stories on interpersonal closeness, corresponding indices were assessed by self-report scales before recalling (Fig. 1*C*). The inclusion of others in the self (IOS) scale is a single item, individuals are asked to pick the pair of circles that best describes the interpersonal relationship (Aron et al., 1992). IOS scale has good reliability and validity to assess the interpersonal closeness (Aron et al., 1992). Several lines of research have proposed that IOS scale has good external validity to measure the interpersonal closeness (Simon et al., 2015; Bentley et al., 2017).

### EEG data acquisition

The neural activity of each participant was simultaneously recorded with an EEG recording system using a 64-channel system (Compumedics NeuroScan) with a sampling rate of 1000 Hz. The electrode cap was positioned following the standard 10–10 international system. Two vertical and two horizontal electrooculogram (EOG) were placed to detect eye-movement artifacts. Impedances were maintained below 10 kΩ.

### Data analysis

#### Behavioral data analysis

The quality of communication between speaker and listener was evaluated using The Listener Recalling stage (see Fig. 1*B*), in which listeners were asked to recall everything they remembered from the stories they heard. Quality of recall was assessed by three raters (following the procedure in Zadbood et al., 2017). The raters first established the rating system by which the quality is principally judged by the detail level of the scene and the accuracy of the narration. Based on this system, they then rated all three stories from the listener independently on the same scale (from 0 to 30). The final quality score of each story was determined by averaging the three raters’ scores on this story. Referred to the method of the similar experimental design (Takahashi et al., 2004), the behavioral index used the contrasts by subtracting the neutral condition from the happy condition and sad condition to assess the specific condition effect. The primary behavioral index “δ recall quality” was computed in the following way: δ recall quality = average recall quality in the emotional group (happy or sad) – the corresponding neutral recall quality. That is, the score of the neutral memory served as a baseline, such that the final scores of emotional memories were subtracted by the mean score of the neutral memories. To evaluate the difference of the behavioral index in sharing quality between happy and sad groups, we conducted an independent-sample *t* test. Cronbach’s αs of 0.91 for the happy video, 0.92 for the sad video, and 0.94 for the neutral video indicate high consistency between the raters.

### EEG data analysis

The EEG raw data were preprocessed and analyzed using the EEGLAB toolbox (version 14.1.0; Delorme and Makeig, 2004) and in-house scripts in MATLAB (R2014a, The MathWorks). EEG data were filtered with a bandpass ranging from 1 to 45 Hz and a notch filter at 50 Hz. Data were re-referenced off-line to an average of the left and right mastoid and downsampled to 250 Hz. EEG data were divided into consecutive epochs of 1000 ms. Eye-movement artifacts were removed with an independent component analysis (ICA) method (Makeig et al., 1996). Signals containing EEG amplitudes greater than ±75 μV were excluded.

EEG data were grouped according to 6 regions for subsequent analysis: (1) frontal (F; AF4, F2, FP2, Fz, Afz, F1, FP1, AF3, F3, F5, F7, F8, F6, F4), (2) frontal-central (FC; Fcz, FC1, FC3, C1, C3, C4, C2, FC4, FC2), (3) parietal (P; CP1, P5, P3, P1, Pz, P2, Cp2, P4, P6), (4) left temporoparietal (left TP; FC5, FT7, C5, T7, TP7, CP5, P7), (5) right temporoparietal (right TP; T6-P8, CP6, TP8, C6, T4-T8, FT8, FC6), and (6) occipital (O; PO3, O1, Poz, Oz, PO4, O_2_). Phase locking value (PLV) is a valid index in EEG brain-to-brain studies (Delaherche et al., 2015; Hu et al., 2018). PLV is a practical method for the direct quantification of frequency-specific synchronization (i.e., transient phase-locking) between two neuroelectric signals and is able to examine the role of neural synchronies as a putative mechanism for long-range neural integration during cognitive tasks. However, compared with the more traditional method of spectral coherence, PLV separates the phase and amplitude components and can be directly interpreted in the framework of neural integration (Lachaux et al., 1999). Thus, the subsequent data were submitted to an IBS analysis known as PLV (Lachaux et al., 1999; Czeszumski et al., 2020). PLV was computed for each pair (i, k) of electrodes for each frequency band according to the following formula:

$${\text{PLV}}_{\text{i},\text{k}}={\text{N}}^{-\text{1}}\left|\sum_{t=1}^{N}exp^{j}{}^{\left(\varphi i(t)-\varphi k(t)\right)}\right|,$$

where *N* represents the number of trials, φ is the phase, | | represents the complex modulus, and i and k indicate the electrode from participants 1 and 2 in a dyad, respectively, where one participant is the speaker and the other is a listener. The PLV ranges from 0 to 1, where PLV equals 1 if the two signals are perfectly synchronized and equals 0 if the two signals are unsynchronized. Phases were extracted using the Hilbert wavelet transform (Schölkopf et al., 2001), and four frequency bands, θ (4–7 Hz), α (8–12 Hz), β (13–30 Hz), and γ (31–48 Hz), were identified as typical frequency ranges in previous studies (Delaherche et al., 2015; Hu et al., 2018). θ Band was expected to observe strongest closeness-related IBS.

Referred to the method of the similar experimental design (Takahashi et al., 2004), the neural index used the contrasts by subtracting the neutral condition from the happy condition and sad condition to assess the specific condition effect. Thus, the present study calculated a δ PLV value in the θ band for each speaker-listener dyad using the equation “δ vPLV = average θ band PLV in the emotional group (happy or sad) – the corresponding neutral average θ band PLV.” We conducted independent-sample *t* tests (Happy vs Sad) on the IBS of speaker-listener dyads to explore the difference of the IBS in sharing stories between happy and sad groups. Differences were considered significant using an electrode-pairs-level threshold of *p *<* *0.05 (Bonferroni-corrected). All PLV analyses focused on the sharing matchup (The Speaker Speaking-The Listener Listening), which represents sharing emotional stories between speaker and listener (Ahn et al., 2018; Chen et al., 2020).

### Correlation between EEG data and behavioral data

To further explore whether the δ value of the IBS was strongly associated with sharing emotional stories, we examined the association between the behavioral (δ recall quality) and neural index (δ PLV in The Speaker Speaking-The Listener Listening).

Moreover, we conducted moderation regression, specifically simple slopes analysis (Aiken and West, 1991), to explore a moderation effect emotion -> PLV × interpersonal closeness. First, we split the file layered by the valence of the emotion (happy/sad). Then, linear regression was used to compare whether *β*-coefficient was significant under the happy group or the sad group.

### Coupling directionality

To estimate the information flow between the speaker and the listener during the Speaker Speaking-Listener Listening matchup, a Granger causality (G-causality) analysis was conducted. According to Granger theory (Granger, 1969), for two given time series X and Y, if the variance of the prediction error for the time series Y at the current time is reduced by including historical information from the time series X in the vector autoregressive model, then the changes in X can be understood to “cause” the changes in Y. The MVGC MATLAB toolbox (Barnett and Seth, 2014) was used to estimate the full multivariate conditional G-causality. The task-related data for each participant were z-scored before G-causality analysis based on the mean and standard deviation of the resting-state signal. A one sample *t* test was used to compare the G-causality of speaker **->** listener with 0.

### Prediction of interpersonal closeness

We conducted a predictive analysis to test whether the IBS of the speaker-listener dyads could predict interpersonal closeness. We conducted an exploratory support vector regression (SVR) analysis, a regression method based on SVM, to explore whether the IBS could predict interpersonal closeness (using the LIBSVM toolbox; Chih-Chung and Chih-Jen, 2011). The response variable here was the listeners’ rating score of the interpersonal closeness. IBS from all electrode pairs was used as the features and a random 70% of the dataset was assigned as a training dataset and the remaining 30% of the data were used as a testing dataset. The model was trained using ε-SVR with the radial basis function (RBF). Based on Hou et al. (2020), the parameter ε was set to 0.01. The other two parameters (C, γ) were optimized using grid search via a fivefold cross-validation method in the training set (Yan et al., 2008). Finally, the Pearson correlation coefficient indicated the prediction accuracy between the actual and predicted values (Kosinski et al., 2013). Collectively, these analyses were conducted to explore whether the IBS of the speaker-listener dyads could predict interpersonal closeness and further to test the generalizability and replicability of our results. To verify the dependency structure of the data, the statistical significance of the correlation between the actual and predicted values was tested by 10,000 permutations.

### Code availability

The sample data and code described in the article is available on-line at https://github.com/XieEnhui/EEG_PLV. The code is available as Extended Data 1. It can be performed using MATLAB (version 2014a) in a Windows 10 system. Full data and codes concerning this study can be available from the authors on request.

10.1523/ENEURO.0245-21.2021.ed1 Extended Data 1 The code used in the study. Download Extended Data 1, ZIP file.

## Results

### Behavioral results

**Figure 2.**
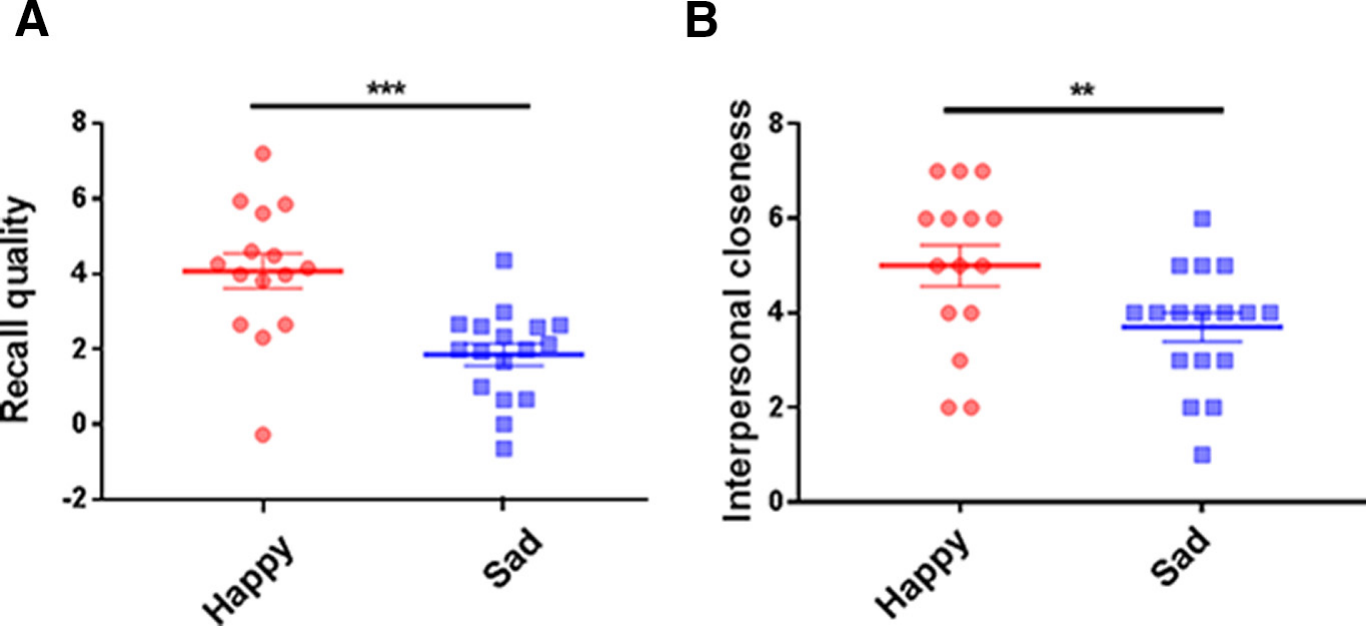
Behavioral results in the happy and sad group. ***A***, The recall quality (δ value) is shown for happy (Happy-Neutral) and sad (Sad-Neutral) emotions. ***B***, The rating of interpersonal closeness is shown for happy (Happy-Neutral) and sad (Sad-Neutral) groups; **p <* *0.01, ***p <* *0.001.

Examination of the recall quality from the happy and sad group revealed significantly better quality in the happy group compared with the sad group (*t*_(30)_ = 4.14, *p *<* *0.001, Cohen’s *d *=* *1.45, independent-sample *t* test; Fig. 2*A*).

In addition, we conducted an independent-sample *t* test on the self-reported interpersonal closeness scores as measured by the IOS. Specifically, the result showed that the happy group had significantly higher scores than the sad group (*t*_(30)_ = 2.91, *p *<* *0.01, Cohen’s *d *=* *0.94; Fig. 2*B*).

### IBS of speaker-listener dyads

Based on this result and previous research (Zheng et al., 2012; Symons et al., 2016), the θ band was used as the band of interest. In the θ band (4–7 Hz), we found that the IBS was significantly higher in the happy group than that in the sad group. The results indicated that the significantly increased IBS between dyads was specific to happy versus sad stories in frontal and temporal regions.

**Figure 3.**
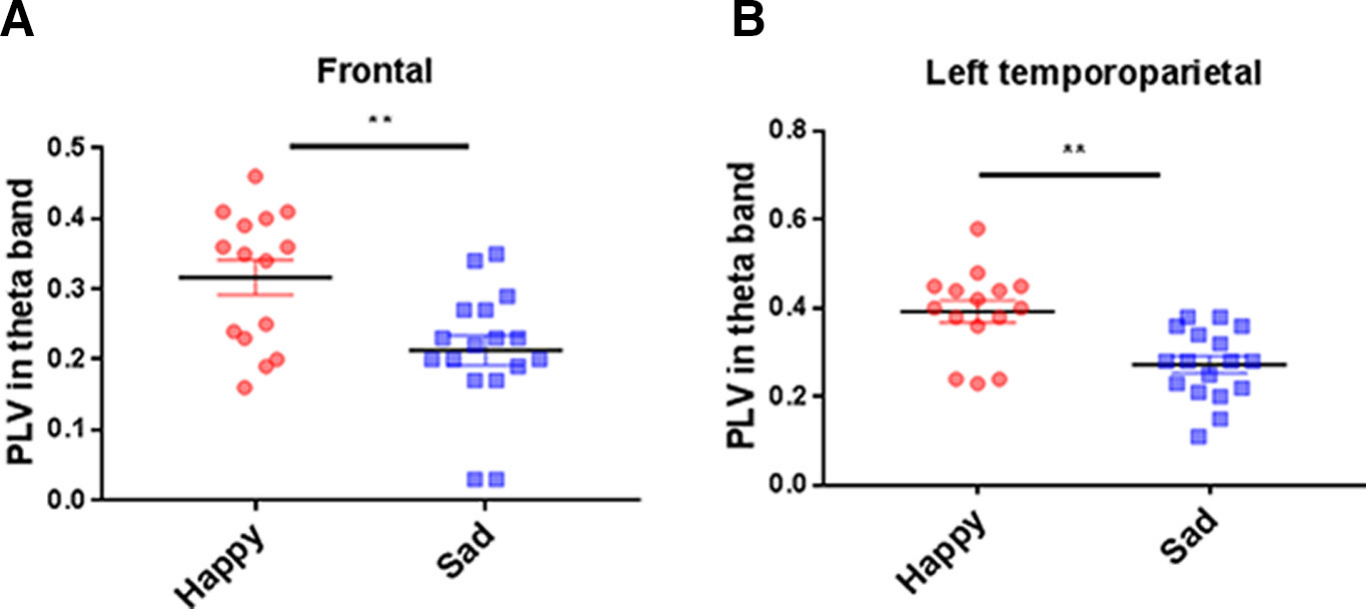
PLV of different groups [happy (Happy-Neutral) and sad (Sad-Neutral)] in sharing matchup (The Speaker Speaking-The Listener Listening). ***A***, The PLV in the θ band at the F site. ***B***, The PLV in the θ band at the F site. All after FDR correction; **p <* *0.01.

IBS during the sharing of emotional stories was measured using task-related PLV. First, we conducted one-sample *t* tests to examine significant differences for the PLVs between the happy/sad group and baseline. As for happy group, the result found significant PLV in the F site (*t*_(14)_ = 12.84, *p *<* *0.004, FDR corrected), FC (*t*_(14)_ = 19.28, *p *=* *0.004, FDR corrected), P (*t*_(14)_ = 14.60, *p < *0.001, FDR corrected), and the left TP site (TP, *t*_(14)_ = 15.84, *p *<* *0.001, FDR corrected) at the θ band. As for sad group, the result found significant PLV in the F site (*t*_(16)_ = 10.14, *p *<* *0.003, FDR corrected), FC (*t*_(16)_ = 12.31, *p *<* *0.003, FDR corrected), P (*t*_(16)_ = 11.60, *p *<* *0.003, FDR corrected), and the left TP site (TP; *t*_(16)_ = 14.13, *p *<* *0.001, FDR corrected) at the θ band. Then, we conducted independent-sample *t* tests to examine significant differences for the PLVs between happy and sad groups. We found that the happy group showed higher PLV than the sad group in the F site (*t*_(30)_ = 3.22, *p *=* *0.003, Cohen’s *d *=* *1.14, FDR corrected; Fig. 3*A*) and the left TP site (*t*_(30)_ = 3.87, *p *=* *0.001, Cohen’s *d *=* *1.36, FDR corrected; Fig. 3*B*) at the θ band. No significant results were found in others regions (see details in Table 2). Previous research has widely suggested that the frontal area is related to emotional communication and language-based interaction (Ahn et al., 2018). Moreover, the left TP is related to the high-level metallization during communications (Samson et al., 2004). Therefore, our further analysis would focus on the PLV in the left TP.

**Table 2:** PLV at different regions and in the θ band between happy and sad group (FDR corrected)

ROIs	PLV of the happy group (mean ± SD)	PLV of the sad group (mean ± SD)	*t*	*d*	df	*p* (corrected)
F	0.32 ± 0.10	0.21 ± 0.09	3.22	1.14	30	0.009
FC	0.47 ± 0.09	0.45 ± 0.15	0.47	0.17	30	0.960
P	0.35 ± 0.09	0.32 ± 0.11	0.81	0.29	30	0.854
Right TP	0.01 ± 0.05	0.00 ± 0.09	0.39	0.14	30	0.839
Left TP	0.39 ± 0.10	0.27 ± 0.08	3.87	1.36	30	0.006
O	0.02 ± 0.10	0.01 ± 0.05	0.94	0.11	30	0.752

F, frontal site; FC, frontal-central site; P, parietal site; right TP, right temporoparietal site; left TP, left temporoparietal site; O, occipital site.

### Neural-behavioral correlation

**Figure 4.**
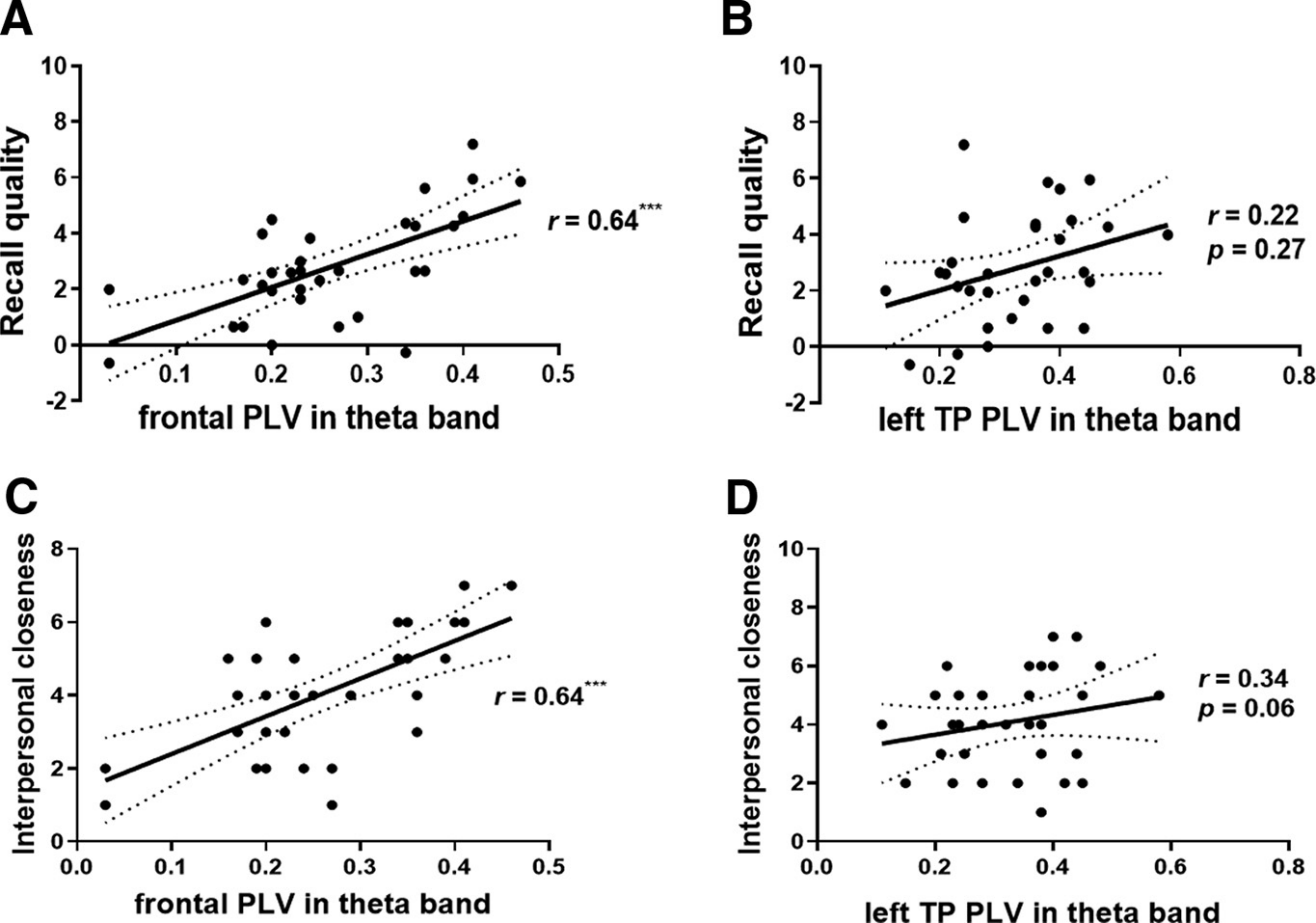
Correlation between behavioral results and frontal IBS. ***A***, Correlation between the recall quality (δ value) and the PLV in the θ band (δ value). The frontal PLV in the θ band positively correlated with the recall quality of dyads. ***B***, We found the left TP PLV in the θ band was not significantly correlated with recall quality of dyads. ***C***, Correlation between interpersonal closeness and the PLV in the θ band. The frontal PLV in the θ band positively correlated with interpersonal closeness of dyads. ***D***, The left TP PLV in the θ band was nonsignificant correlated with interpersonal closeness of dyads; *p <* *0.05, ***p <* *0.001.

We examined the association between the recall quality and PLV in the θ band. Results revealed that the recall quality showed a significant, positive association with the frontal PLV in the θ band (*r*_(32)_ = 0.64, *p *<* *0.001; Fig. 4*A*). We found no significant association with the left TP PLV in the θ band (*r*_(32)_ = 0.22, *p *=* *0.27; Fig. 4*B*). Self-reported interpersonal closeness showed a significant and positive association with the frontal PLV in the θ band (*r*_(32)_ = 0.64, *p *<* *0.001; Fig. 4*C*). We found no significant association with the left TP PLV in the θ band (*r*_(32)_ = 0.34, *p *=* *0.06; Fig. 4*D*).

A moderation effect was estimated, results revealed that the *β*-coefficient was significant in the happy group (*β* = 0.68, *SE *=* *3.74, *t *=* *3.36, *p_happy_* < 0.01), but the *β*-coefficient was nonsignificant in the sad group (*β* = 0.45, *SE *=* *3.77, *t *=* *1.98, *p_sad_* = 0.07). Thus, PLV × self-reported interpersonal closeness was moderated by the valence of emotion.

### Coupling directionality

The G-causality analysis was used to measure the directional information flow (i.e., speaker **->** listener). The one sample *t* test showed that there was a significant difference between 0 and the G-causality of speaker **->** listener in The Speaker Speaking-The Listener Listening matchup (*t*_(31)_ = 13.35, *p *<* *0.001). To sum up, the result indicated that the information could only be transmitted from the speaker to the listener and the speaker is a significant predictor of future values of the listener.

### Prediction of interpersonal closeness

**Figure 5.**
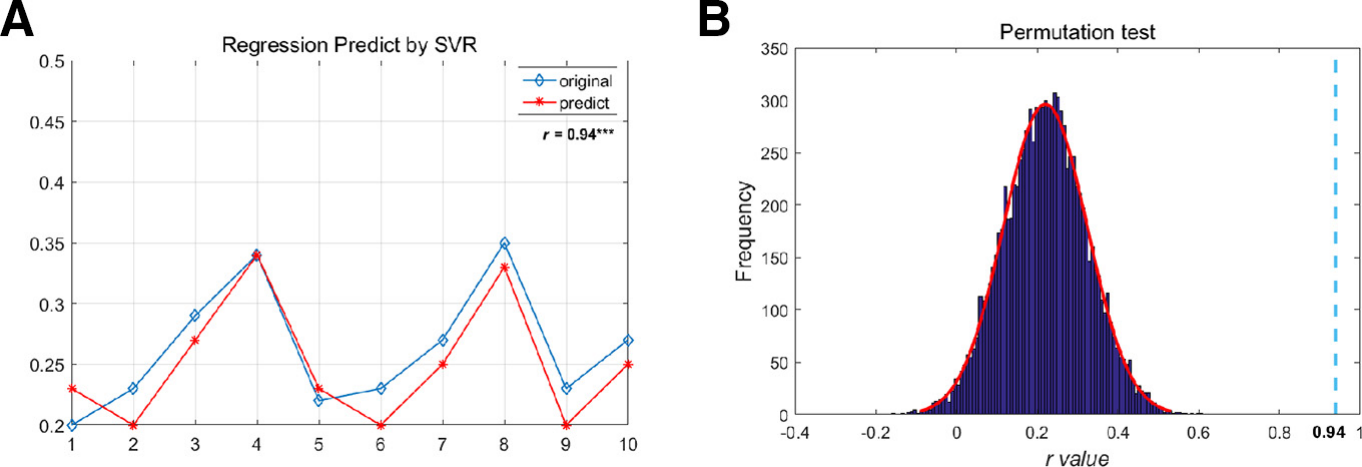
Frontal IBS in the θ band can effectively predict interpersonal closeness. ***A***, Regression predicted by SVR between real value and predict value. ***B***, The *r* value is calculated as a metric of prediction interpersonal closeness. The significance level (threshold at *p* < 0.05) is calculated by comparing the *r* value from the correct labels (dotted line) with 10,000 randomization samples with shuffled labels (blue bars); ***p <* *0.001.

Evaluating whether IBS as a neural indicator in the present study can be used to predict the interpersonal closeness between speaker-listener dyads, we used the SVR analysis. The results showed that the correlation coefficient between the actual and predicted interpersonal closeness of the testing dataset was ∼0.98, *p *<* *0.001 (Fig. 5*A*). As expected, the real *r* values were significantly greater than the majority of the 10,000 permuted *r* values (Fig. 5*B*). Thus, the IBS in the frontal region as the specific neural-behavioral related index can predict the interpersonal closeness between speaker-listener dyads.

## Discussion

In the present study, we explored (1) the association between sharing stories and interpersonal closeness and (2), its underlying neural correlates (3) and, the different roles of happy and sad emotions (i.e., Happy vs Sad) in sharing stories and building interpersonal closeness. As expected, the present study revealed that the behavioral index (the quality of recall after hearing an emotional story) was positively associated with interpersonal closeness in both happy and sad groups. Compared with the sad group, the happy group showed better recall quality and reported higher interpersonal closeness. Moreover, higher task-related IBS was found for the happy group. Within the happy group, sharing stories moderated the IBS and thus promoted interpersonal closeness. Finally, the F site IBS can be used to predict interpersonal closeness. The aforementioned results are discussed in detail as follows.

Our results showed a positive association between sharing emotional stories and interpersonal closeness at the behavioral level. Further, in the light of EASI, humans tend to synchronize with each other’s behavior (McCarthy and Duck, 1976; Dubois et al., 2016) and physiological states (Konvalinka et al., 2010) during emotional expression, our results suggested that sharing happy and sad stories can facilitate interpersonal closeness. Consistent with previous studies, we found that sharing happy stories was more likely to be transferred and received than sharing sad stories (Piotroski et al., 2015), we also found that the happy group showed a better recall quality compared with the sad group and led to higher interpersonal closeness. Moreover, initial behavioral studies have shown that participants preferred positive experiences on social media (Gable et al., 2004; Dorethy et al., 2014; Pounders et al., 2016), and thus sharing happy stories may represent a positive image and be good for building interpersonal relationships with strangers (Birnbaum et al., 2020).

Examining the cognitive and neural processes involved in social interaction behaviors hinges on investigating brain-to-brain synchronization during social interaction. “Two-person neuroscience” in sharing stories has higher ecological validity than single-brain recoding because “two-person neuroscience” is closer to the real-life interactions (García and Ibáñez, 2014; Joy et al., 2018; Redcay and Schilbach, 2019). Moreover, brain-to-brain studies have been widely accepted to unveil the interpersonal neural correlates in the context of social interactions (Lu et al., 2019; Chen et al., 2020). Based on the neuroimaging studies, sharing stories may be inherently reflected on the neural level (Tamir and Mitchell, 2012; Berger, 2014), and comprehension of narrations was driven by the neural similarity between the speaker and the listener (Silbert et al., 2014; Nguyen et al., 2019). A similar understanding of stories during interpersonal interaction led to enhanced IBS which represented the higher neural similarity of speaker-listener dyads (Hasson et al., 2012; Jiang et al., 2015; Nozawa et al., 2016; Chen et al., 2020). Therefore, the present study used brain-to-brain recording to evaluate the dynamic neural interaction between the speaker and the listener, revealing a brain-to-brain interaction pattern in the process of sharing stories (the sharing matchup). Referred to previous emotional communication brain-to-brain studies and audio brain-to-brain studies (Smirnov et al., 2019; Hou et al., 2020), the present study was not real-time interaction. Consistent with previous studies (Tamir et al., 2015; Kulahci and Quinn, 2019), our findings suggested that high IBS levels represented a high-level story comprehension of sharing stories and that was essential to increase interpersonal closeness between individuals.

We found significant IBS between speaker and listener in the frontal cortex during interaction in the θ band, consistent with previous studies which indicated that θ band was associated with emotion and memory encoding (Klimesch et al., 1996; Ding, 2010; Symons et al., 2016). Previous brain-to-brain studies have found strong interpersonal neural synchronization in the frontal cortex using the interactive paradigm involving verbal communication (Ahn et al., 2018; Bohan et al., 2018). Moreover, prior studies have uncovered that the frontal cortex critically contributes to recognizing emotions and encoding information (Abrams et al., 2011; De Borst et al., 2016). Therefore, our finding is consistent with previous findings, demonstrating that the frontal cortex was correlated with establishing a frame of emotional information.

Our results indicated the valence of emotion played a moderating role between IBS and interpersonal closeness. A recent study has shown that the neural synchronization between the speaker and the listener was associated with emotional features of stories and that the neural synchronization created a tacit understanding between the speaker and the listener, facilitating communication and improving interpersonal relationships (Smirnov et al., 2019). It is worth noting that only the happy emotion (relative to neutral) played a moderating role in enhancing IBS. Although the theory of EASI proposes that emotional expression will increase mutual understanding between individuals, compared with positive emotional expression, the effect of negative emotional expression is subtle (Dubois et al., 2016). Individuals prefer to share positive self-related details (i.e., happy stories about the videos you watched) in the presence of strangers and they also wish to transfer ideal images (Tamir and Mitchell, 2012; Baek et al., 2017). On the neural level, our result further evaluated the key role of different emotions (i.e., happy and sad) in the mediation effect of sharing stories on interpersonal closeness. To sum up, our results supported that sharing happy stories is more helpful in enhancing speaker-listener interaction.

Our GCA results further showed that there was a significant directionality of the enhanced IBS between the speaker and the listener, implying that the speaker was a significant predictor of future values of the listener above and beyond past values of the listener. Our findings were consistent with previous studies in unilateral communication or unilateral sharing, the speaker owns more information than the listener (Tworzydło, 2016). Based on the verbal cues of the speaker, the listener would frame the information, fill in the content, and adjust the content during the dynamic interactive process (Chen et al., 2017; Zadbood et al., 2017; Nguyen et al., 2019). In line with previous findings, listeners would perform as followers during sharing emotional stories and this performance is influenced by the speaker (Stephens et al., 2010; Jiang et al., 2015; Bohan et al., 2018). Therefore, the directionality of IBS in our study highlighted the point that sharing emotional stories were dominated by the speaker.

Our results revealed the predictive effect of frontal IBS for interpersonal closeness through SVR. These findings were in line with recent studies revealing that synchronized brain activity served as a reliable neural-classification feature (Cohen et al., 2018; Hou et al., 2020; Pan et al., 2020). Moreover, a growing number of studies have used the combination of machine learning and the IBS measurement in social neuroscience, so we considered more features, such as the time-frequency neural features from single-trial event-related spectral perturbation (ERSP) patterns (Makeig et al., 2004; Chung et al., 2015).

The present study had several limitations. First of all, the present study focused on specific happy and sad videos. Although the present study demonstrated that there is no difference between videos except for their valence, so the different effects on sharing stories are not driven by other variables related to the stories (e.g., recall duration, vividness, social context, etc.), the generalizability of the present results to more emotional videos needs to be examined in future studies. Second, spatial resolution is restricted in EEG, which is distributed on the skull and scalp (Hedrich et al., 2017), limiting measurements to specific areas during sharing emotional stories of speaker-listener dyads. Our findings indicated that frontal and temporal cortices were important in sharing emotional stories. Although the ventromedial PFC (VMPFC) and anterior cingulate cortex (ACC) play a crucial role in sharing emotional information activities (Killgore et al., 2013), EEG is unable to measure these two areas. Finally, the exploratory SVR predictive analysis is constrained by relative sample size (although our sample size is similar to those reported in previous classification and prediction analyses based on brain-to-brain coupling data; Jiang et al., 2012; Dai et al., 2018; Pan et al., 2020). Future replications are encouraged to consolidate the current findings by increasing both the sample size and the number of testing blocks.

In conclusion, the present study showed that sharing both happy and sad stories could increase interpersonal closeness between individuals. Moreover, findings at the neural level suggested that only sharing happy stories moderated the frontal IBS and thus promoted interpersonal closeness. These insights contribute to a deeper understanding of the neural correlates of sharing different emotional stories with interpersonal closeness. Future research may explore the neural mechanism of sharing stories by using IBS as an effective neural indicator.

## Synthesis

Reviewing Editor: Macià Buades-Rotger, University of Luebeck Decisions are customarily a result of the Reviewing Editor and the peer reviewers coming together and discussing their recommendations until a consensus is reached. When revisions are invited, a fact-based synthesis statement explaining their decision and outlining what is needed to prepare a revision will be listed below. The following reviewer(s) agreed to reveal their identity: Shannon Burns.

Dear authors,

Thanks for your work on interbrain synchrony and interpersonal closeness. While most of our concerns have been successfully addressed, I cannot recommend the manuscript for publication until some last substantial changes are incorporated. Two general themes must be addressed: a) the manuscript must be proofread by a proficient English speaker as the language is overall poor (see e.g., the minor concerns below); and b) the authors should include content from the rebuttal in the actual manuscript for the sake of transparency. In the following I list these issues in more detail:

Major concerns:

- Page 4: the introduction still fails to pinpoint the exact research question and how it fits in the literature. How, precisely, does this study inform the literature? What do we know not that we did not know earlier? Furthermore, the authors should briefly describe where in the brain and in which frequency bands they expected to observe strongest closeness-related IBS.

- Page 6: the authors write “we gave more evidence that the videos did not significantly differ in the emotional arousal, the amount of social content, and vividness, but only in the valence", yet they do not provide any actual results. Please provide the statistics reported in the rebuttal as well as the demographics of the 10 additional valence/arousal raters. Also, please rephrase the paragraph added on page 5 more clearly. The following part is especially confusing: “Results identified the valence of happy video is > 7 (happy), neutral video is 4 < rating < 6 (neutral), and sad video is < 3 (sad).” What does this mean? Is this really “identified by the results” or is it simply an arbitrary scoring?

- Also on page 6, the authors write “The emotional arousal of spoken recall recordings was evaluated by listeners (range from 1 to 7). The average emotional arousal of the happy story recording was 6, the average emotional arousal of the neutral story recording was 5, and the average emotional arousal of the sad story recording was 6.” Which listeners are these? Please specify.

- Page 10: the authors should include the justification for using PLV instead of spectral coherence in the manuscript. That is, the reply on page 8 of the rebuttal should be on the manuscript.

- Page 15: the authors should provide more information about the regression model reported.

- Please provide a data availability statement and upload all data and code in a public repository so results can be reproduced. If this is not possible, include it in the availability statement.

- What is the scale for the interpersonal closeness measure? What evidence is there of its validity? Please provide some more details.

Minor concerns:

This is by no means an exhaustive list but it should illustrate the need for proofreading:

- Lines 95-102: listeners do not “evoke", they “experience” emotions. The ones doing the evoking are the speakers.

- Line 319: “The result found that”. Results do not find anything, they are found by someone.

- Line 331: “A subsequent correlation analysis examined the association”. The correlation analysis did not examine anything, the authors did.

-
